# Direct transmission of the cat flea (*Ctenocephalides felis*) between cats exhibiting social behaviour

**DOI:** 10.1051/parasite/2013050

**Published:** 2013-12-06

**Authors:** Michel Franc, Émilie Bouhsira, Frédéric Beugnet

**Affiliations:** 1 Université de Toulouse, INP, ENVT, F-3107 École Nationale Vétérinaire de Toulouse 23 chemin des Capelles 31079 Toulouse France; 2 Merial 29 Avenue Tony Garnier 69007 Lyon France

**Keywords:** *Ctenocephalides felis felis*, Cat, Infestation, Biology

## Abstract

A study design was created to assess the potential for fleas to infest cats directly from other cats. In the first experiment, six cats were infested with 100 fleas each and then immediately put in contact with six flea-free cats for 24 h. After removal of all fleas the study was repeated and the contact between cats lasted 48 h. The total numbers of fleas recovered out of the 600 fleas deposited on the 6 donor cats after each infestation were 499 and 486 at 24 h and 48 h respectively. At 1 h post-contact, five fleas were found on the receiver cats, with three cats having one flea and one cat, two fleas. The number of fleas recovered on receiver cats increased towards the end of the study. At 24 h, 20% of the fleas were found on the receiver cats, and at 48 h, 23%. In a second experiment, the six flea-free cats were put in contact with the six donor cats which were each infested by 100 fleas 48 h before. Fewer fleas were found on the receiver cats (*n* = 15), representing 3.8% of all fleas recovered (*n* = 403). All the observed fleas had fed. The fleas collected on receiving cats comprised 10 males and 5 females, and 4 of the 5 females were engorged and contained eggs. The fleas collected on donor cats comprised 153 males and 235 females, they were all fed and all females contained eggs. This experiment demonstrated that gravid female fleas have a tendency to become permanently but not exclusively parasitic. Nevertheless, a few can change their cat host in as little as 1 h, which may play a role in the rapid introduction of a new flea population into a cat environment.

## Abstract (fr)

Une étude a été conçue pour évaluer la capacité des puces à infester directement des chats à partir d’autres chats. Dans un premier dispositif expérimental, six chats ont été infestés chacun par 100 puces et ont été mis immédiatement en contact avec 6 autres chats non infestés, pour une durée de 24 heures. Cette expérience a été renouvelée à l’identique mais en gardant les chats ensemble pendant 48 heures. Le nombre total de puces récupérées à partir des 600 puces déposées à l’origine sur les 6 chats donneurs a été de 499 à 24 heures et 486 à 48 heures. Une heure après contact, 5 puces ont été trouvées sur les chat receveurs, trois chats hébergeant une puce et un chat deux puces. Le nombre de puces récupérées sur les chats receveurs a augmenté tout au long de l’étude. A 24 heures, 20% des puces ont été retrouvées sur les chats receveurs, 23% à 48 heures. Dans une deuxième expérience, les 6 chats receveurs ont été mis en contact avec les chats donneurs qui avaient été préalablement infestés par 100 puces chacun, 48 heures avant. Très peu de puces ont été retrouvées sur les chats receveurs (*n* = 15), représentant 3,8% de la totalité des puces collectées (*n* = 403). Toutes les puces s’étaient nourries. Les puces collectées sur les chats receveurs comprenaient 10 mâles et 5 femelles, 4 des 5 femelles étaient gorgées et contenaient des œufs. Les puces collectées sur les chats donneurs comprenaient 153 mâles et 235 femelles, toutes les puces s’étaient nourries et toutes les femelles contenaient des oeufs. Cette étude a démontré que les puces gravides ont tendance à devenir des parasites permanents mais non exclusifs. Néanmoins, certaines peuvent changer d’hôtes en un temps aussi bref qu’une heure, ce qui peut jouer un rôle dans l’introduction rapide de nouvelles populations de puces dans l’environnement de chats.

## Introduction

The cat flea, *Ctenocephalides felis felis* (Bouché, 1835) (Siphonaptera, Pulicidae) [1], is the most prevalent ectoparasite of cats in the world [8]. It is the cause of dermatological signs including pruritus, squamoseness, hairloss and the appearance of flea allergy dermatitis in some pets [7]. The cat flea is also a vector of several pathogens including *Bartonella henselae* and *Rickettsia felis*, and the intermediate host of the tapeworm *Dipylidium caninum* [3]. It is acknowledged that the main sources of cat fleas for pets are the newly emerged fleas present in the environment (households, gardens) [9, 12]. The pet’s environment contains pre-emerged fleas in pupae. Following emergence stimuli, the new fleas hatch and need to find their host quickly. The females and males will take their first blood meal within 2 h and then 4–10 times per day. They mate during the first 24 h and the females will start to lay eggs within 24–48 h after having infested the host. They lay around 30 eggs per day during 2 weeks and they live for 2–3 weeks. The eggs fall off the host within 2–3 h. They hatch in a few days. Three larval stages will evolve within 10–14 days if temperature and humidity conditions are optimum and then finally form the pupae. The pupae are the resistant stage in the environment, they can survive for around 6 months waiting for a host [2, 7, 9]. It is accepted that the major source of new fleas comes from the emerging pupae present in the environment, nevertheless, taking into account the social contacts between cats, it can be hypothesised that the direct transfer of mature gravid fleas from one cat to another may not be negligible and could lead to the introduction of a new flea population in a cat’s environment. This was firstly assessed by Rust in 1994 [10] on two cats with preliminary results demonstrating the possibility of flea movements between cats. The aim of this study was to better assess the rate of flea transmission between cats living together in an environment simulating natural behaviour, including feeding, sleeping and playing.

## Materials and methods

This study was conducted in compliance with Good Scientific Practices in the Parasitology Contract Research Organization Facility of Toulouse Veterinary Faculty, approved by the French Agricultural Ministry and the European Medicinal Agency (Agreement C3155511), under the supervision of Professor Michel Franc (Agreement 31-08-555-04). Eighteen adult cats (>6 months) originated from the facility were first included in the study. These cats were European shorthair cats purchased from commercial research catteries (Charles River and Harlan NL). They were born between April 2002 and November 2009. They were identified by the last four numbers of their electronic microchip. All cats had a physical examination on Day-14 to ensure a healthy status. A complete combing lasting at least 7 min ensured that the cats were flea-free prior to initiation of the study.

**Figure 1.**
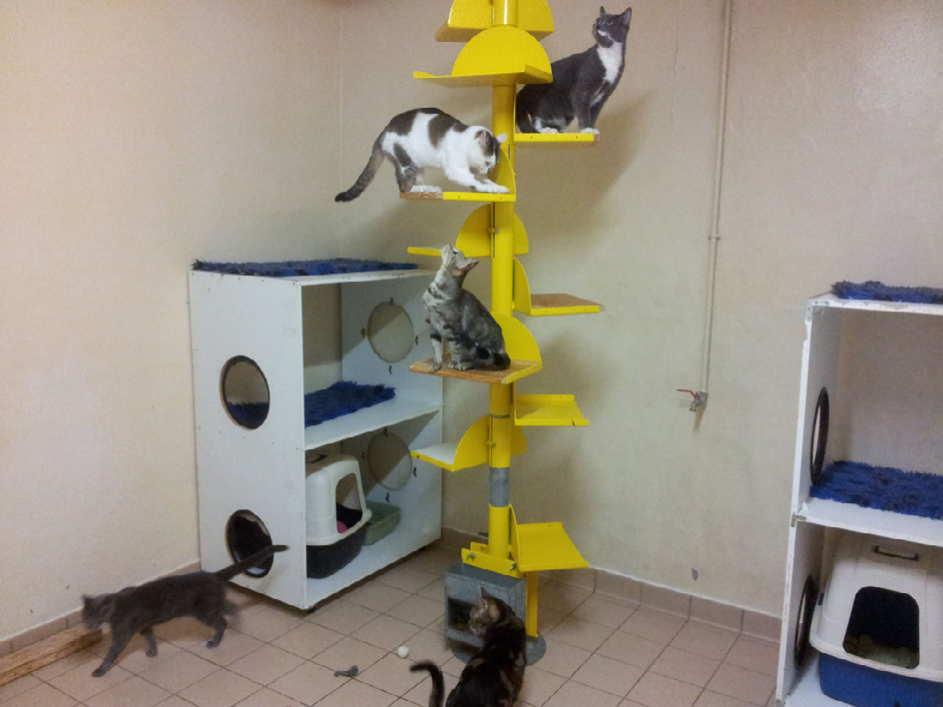
Experimental cat room.

On Day-14, the 18 cats were grouped in a 20 m^2^ room simulating a natural environment, including games and resting areas in order to socialise them (Figure 1). The room was maintained under environmental controlled conditions: temperature 18–21 °C, ambient air exchanged 14 times per hour, lighting 12 h light/12 h dark; humidity was not controlled.

Before the acclimation period, and before cats being introduced in the experimental rooms, all rooms were vacuumed and then cleaned out with hot water under pressure and hot steam. A delay of 1 week was respected before the introduction of cats. Then after the start of the study, the rooms were vacuumed and cleaned with water and soap and the litter trays changed every day.

On Day-10, all cats were infested with 100 *Ctenocephalides felis* adult fleas. The fleas used were *Ctenocephalides felis felis* belonging to the Toulouse Vet School laboratory strain. This strain was obtained from a laboratory-reared colony originating from a wild strain harvested from a cat and maintained on cats under laboratory conditions since 1990. The morphology of the fleas is regularly checked and confirmed to be *Ctenocephalides felis felis*. They have also been checked via quantitative molecular biology tools such as qPCR. DNA of *C. felis* is detected by amplification of a fragment of *C. felis* 18s rDNA derived from an available partial sequence of *C. felis* 18S rDNA in GenBank (Accession Number AF 136859).

The cats were observed carefully for 3 days to detect any excessive groomers or cats whose social attitude changed when they were infested with fleas (likely cats developing Flea Allergy Dermatitis), as well as any non-social cats.

On Day-7, all cats were flea-combed and 6 cats were removed, including the excessive groomers, non-social cats and cats with the lowest flea numbers. The 12 remaining cats were selected for the study. They were ranked by pair and allocated based on flea counts into group 1 and 2. The sex, age and hair length were not considered important for allocation purposes.

Finally two groups of six cats were obtained. The cats were not treated with ectoparasiticides or insect growth regulators (either topical or systemic) within 3 months of the start of the study.

## Experiment 1 (Table 1)

The six cats in group 1 were each infested with 100 fleas on Day 0. The six cats in group 2 remained uninfested.

**Table 1.** Schedule of the first experiment.

Study Day	Infestation	Event
Day 0	Flea infestation	Infest each cat from Group 1 with 100 (±5) *C. felis* fleas
		Put all cats (group 1 and group 2) in the room
+1 h		Comb and remove fleas from all group 2 cats, return the cats to the room
+6 h		Comb and remove fleas from all group 2 cats, return the cats to the room
+24 h (Day 1)		Comb and remove fleas from all cats (group 1 and 2), return the cats to the room
Day 2 (same week)	Second flea infestation	Re-infest each cat from group 1 with 100 (±5) fleas. Leave all cats in the room
+2 h		Comb and remove fleas from all group 2 cats, return the cats to the room
+12 h		Comb and remove fleas from all group 2 cats, return the cats to the room
+48 h (Day 6)		Comb and remove fleas from all cats (group 1 and 2)

The cats in group 1 were placed in the experimental 20 m^2^ room with space, games, resting areas and food (Figure 1). The six flea-free cats were introduced 15 min later into the room.

The group 2 cats were combed at 1 h, 6 h and 24 h, their fleas removed, and then the cats were placed back into the experimental room after each count. Twenty-four hours after the start, the fleas were also removed and counted from the cats in group 1.

The experiment was duplicated a second time during the same week. The room was cleaned and the cats were combed in order to ensure a flea-free status before the experiment started. The group 1 cats were again infested with 100 fleas each. The group 2 cats were mixed with them and then assessed for fleas at 2 h, 12 h and 48 h afterwards.

## Experiment 2 (Table 2)

In the first experiment, the cats from group 1 were infested and then put together with the flea-free cats. Therefore the fleas were still young adult fleas, most probably fed, but still not gravid and not ready to lay eggs.

**Table 2.** Schedule of the second experiment.

Study Day	Event
Day 0	Infest each cat of Group 1 with 100 (±5) *C. felis* fleas
	Put the cats together in the large room
Day 2 (48 h)	Introduce the six “receiver” flea-free cats (Group 2)
+1 h	Comb and remove fleas from group 2 cats, return the cats to the room
	Check the fleas to determine the number of males/females, feeding status and if the females contain eggs
+6 h	Comb and remove fleas from group 2 cats, return the cats to the room
	Look at the fleas to determine the number of males/females, if they are engorged and if the female contains eggs
+24 h (Day 3)	Comb and remove fleas from all cats (group 1 and 2)
	Look at the combed fleas to determine the number of males/females, if they are engorged and if the females contain eggs

In the second experiment, the fleas were left on the infested cats for 48 h before introducing the six flea-free cats from group 2. Based on flea biology, it could be assumed that the female fleas would be fed and gravid. The majority of fleas bites had occurred between 40 min and 1 h after infesting their host [2, 9]. They reproduce during the first 24 h and start to lay eggs between 36 and 48 h after infestation [8].

The six cats from group 1 were infested with 100 fleas each on Day 0 while the six cats from group 2 remained uninfested. The group 1 cats were placed in the cleaned experimental room, offered space, games, resting areas and food. The six flea-free cats from group 2 were introduced into the room 48 h later.

The group 2 cats were checked for fleas 1 h, 6 h and 24 h after being introduced into the room. The fleas from the group 1 cats were also removed at the latest time point. All the removed fleas were observed microscopically in lactophenol to identify the sex (male versus female), feeding status (fed/unfed) and presence or absence of eggs.

Statistical analysis was done using Chi-square test.

## Results

### Experiment 1

Two successive flea infestations were done during the first week of the experiment to allow several time points to be used (Table 3). The total numbers of fleas recovered out of the 600 deposited on the six donor cats at each infestation were 499 and 486 at 24 h and 48 h respectively. This represents recovery rates of 83.12% at 24 h and 81% at 48 h.

**Table 3.** Results of flea counts on cats in contact with cats infested by fleas 15 min previously.

		Groups	First flea infestation on donor cats			Second flea infestation on donor cats		
CAT ID	No. of fleas	Donor cats	1 h	6 h	24 h	2 h	12 h	48 h
3996	100	D	/	/	78	/	/	93
6533	100	D	/	/	57	/	/	82
2823	100	D	/	/	84	/	/	62
9165	100	D	/	/	65	/	/	53
6532	100	D	/	/	55	/	/	47
4117	100	D	/	/	60	/	/	38
		Sum	/	/	399	/	/	375
		Receiver cats	1 h	6 h	24 h	2 h	12 h	48 h
8261		R	2	5	15	4	5	14
7138		R	1	2	29	2	7	18
6502		R	1	4	21	3	5	20
4644		R	0	6	8	5	6	9
3812		R	0	0	1	2	6	10
6506		R	1	7	26	10	15	40
		Sum	5	24	100	26	44	111

At 1 h, five fleas were already found on the receiver cats, three cats having one flea and one cat having two fleas.

The number of fleas recovered from the receiver cats increased towards the end of the study (Table 3).

At 2 h, all receiver cats were found to be flea infested. The difference in flea counts was not significantly different between the counts done at 24 and 48 h (Chi-square test).

At 24 h, 20% of the fleas were found on the receiver cats, 22.8% at 48 h, with a respective average of 16.7 and 18.5 fleas on receiver cats versus an average of 66.5 and 62.5 fleas on the donor cats at 24 h and 48 h.

### Experiment 2

Out of the 600 fleas deposited on six cats, 403 fleas were recovered 3 days after infestation (67.16%), 388 on the donor cats and 15 on the naive cats. One hundred fleas from the infesting population were checked for sex ratio. The batch contained 58 females and 42 males, meaning a sex ratio F/M of 1.38.

Compared to experiment 1, a lower number of fleas was found on the receiver cats (*n* = 15) representing 3.7% of all fleas recovered (*n* = 405) (Table 4). All the observed fleas had fed. The collected fleas comprised 10 males and 5 females, and most interestingly four of the five females were engorged and contained eggs. This demonstrated the capacity of mature gravid fleas to infest new cats in as little as 24 h. The sex ratio in this group was 0.5.

**Table 4.** Results of flea counts on cats in contact with cats infested by fleas 48 h previously.

		Groups	1 h	6 h	24 h	Total
CAT ID	No. of fleas	Donor cats				
3996	100	D	/	/	23 M 32 F	55
6533	100	D	/	/	21 M 48 F	69
2823	100	D	/	/	18 M 37 F	55
9165	100	D	/	/	31 M 30 F	61
6532	100	D	/	/	20 M 35 F	55
4117	100	D	/	/	40 M 53 F	93
		Sum	/	/	153 M 235 F	388
		Receiver cats				
8261		R	1 fed F	1 fed M	0	2
7138		R	1 fed M	1 fed M	1 fed F eggs	3
6502		R	1 fed F eggs	0	1 fed M	2
4644		R	1 fed M	1 fed M	1 fed M	3
3812		R	0	1 fed F eggs	0	2
6506		R	0	2 fed M	1 fed M	3
					1 fed F eggs	
		Sum	4	6	5	15

Fed/unfed; F = Female; M = Male; Eggs = presence of eggs inside female.

Out of the 388 fleas recovered from the infested cats (group 2), 153 were male and 235 were female (sex ratio F/M = 1.53). All females were gravid.

The sex ratio in the originally infested cats was not significantly different at the time of infestation (1.58) and after 24 h (1.53). On the other hand, there was a significant difference at 24 h between the six donor cats (sex ratio = 1.53) and the six receiver cats (sex ratio = 0.5) (*p* < 0.05) indicating that male fleas were more likely to change their host than females.

## Discussion

Cats usually display social behaviour when living in the same neighbourhood. They play and feed together, and of course also have skin contact during sexual activity. The first experiment aimed to prove that fleas could be transferred quickly and in a non-negligible percentage between cats with a similar lifestyle. These fleas were fed but not mature female fleas.

We demonstrated in the first experiment that within the first hour, fleas had already moved from infested cats to flea-free cats. The percentage of fleas having changed their host was at least 20% after one day. We did not assess movement between the infested cats, which could also have occurred. The decrease in the speed of transfer after 24 h may be due to several factors, including the increase of grooming behaviour in the infested cats but also the maturation of the fleas becoming engorged and gravid. This first experiment indicates that cats that have recently been infested and have contact with others facilitate the easy transfer of fleas.

In the second experiment all fleas matured on the infested cats before the contact phase with flea-free cats. The number of recovered fleas significantly decreased in the second experiment (403/600, 67.16%) compared to the first one (83.12% at 24 h) (*p* < 0.05). This is probably due to an increase in the grooming activity with time. A study conducted in 1998 showed that cats were able to groom and ingest up to 17.6% of their fleas each day, leading to a lifespan of about 8 days for a flea [8]. In our second experiment, the infestation of the introduced flea-free cats occurred as soon as 1 h after contact but at a lower rate than in the first experiment. It can be hypothesised that mature fleas have a greater tendency to be sedentary and to remain permanent parasites. This transfer of fleas was indirectly demonstrated by Bradbury and Lappin (2010) when studying the infections of cats by *Bartonella* [4]. In their study, the *Bartonella*-infected cats were placed in a cage and infested by fleas. Another group of cats was placed in a cage alongside. The cats could not have any contact but the meshed walls and the bottom of the cages allowed some passage for fleas. At the end of the trial, the naive cats were flea-infested and *Bartonella* positive. This design showed that fleas were able to fall off their host, to walk on the floor or through the mesh and pass into the next cage. In a field study conducted in Tampa, Florida, Dryden et al. (2011) were able to collect fed fleas and mature female fleas in flea traps [5]. Thirty-four percent of the fleas found in traps were fed and 1.16% of those fed fleas were females containing eggs, demonstrating that mature fleas can be found on the floor. In his preliminary study using two cats and artificially coloured fleas, Rust demonstrated that interhost movement occurs and, similarly to our second experiment, that gravid female fleas move less than males [10]. He found that up to 15% of fleas may change their host which is close to what we observed in the first experiment.

In our study, the flea movement was probably direct, without passage in the environment, but this cannot be excluded. In the second experiment, all transferred fleas were found fed and almost all the females already contained eggs. Even if the rate of transfer was low in 24 h, it allowed mature males and females to infest new cats and to initiate a new flea population immediately. The comparison of the sex ratio between the groups indicated that males are more likely to change their host than mature females. Based on the known biology and the demonstrated role of newly emerged fleas as the main source of infestation, our study demonstrated that the direct infestation from cats to cats does exist and can be considered as a possible source of introduction in a new environment [10, 12]. This modality raises the importance of the use of a combination of insecticide and Insect Growth Regulator (IGR) on cats for the control of new flea infestations [2, 11]. In a situation where infestation by a gravid female flea occurs, its eggs would be laid before the death of the flea due to the adulticide, contrary to what is seen with new unfed fleas. The IGR would then ensure that the eggs were non-viable, blocking the evolution of those eggs and maintaining control of the environmental contamination, which has been shown by Franc et al. (2007) using a gravid flea infestation model [6].
